# *N*-n-butyl haloperidol iodide ameliorates hypoxia/reoxygenation injury through modulating the LKB1/AMPK/ROS pathway in cardiac microvascular endothelial cells

**DOI:** 10.18632/oncotarget.9186

**Published:** 2016-05-05

**Authors:** Binger Lu, Bin Wang, Shuping Zhong, Yanmei Zhang, Fenfei Gao, Yicun Chen, Fuchun Zheng, Ganggang Shi

**Affiliations:** ^1^ Department of Pharmacy, The First Affiliated Hospital, Shantou University Medical College, Shantou 515041, Guangdong, China; ^2^ Department of Biochemistry and Molecular Biology, University of Southern California, Los Angeles, California 90033, USA; ^3^ Department of Pharmacology, Shantou University Medical College, Shantou 515041, Guangdong, China; ^4^ Department of Cardiovascular Diseases, The First Affiliated Hospital, Shantou University Medical College, Shantou 515041, Guangdong, China

**Keywords:** N-n-butyl haloperidol iodide, LKB1/AMPK/ROS, hypoxia/reoxygenation, cardiac microvascular endothelial cells

## Abstract

Endothelial cells are highly sensitive to hypoxia and contribute to myocardial ischemia/reperfusion injury. We have reported that *N*-n-butyl haloperidol iodide (F_2_) can attenuate hypoxia/reoxygenation (H/R) injury in cardiac microvascular endothelial cells (CMECs). However, the molecular mechanisms remain unclear. Neonatal rat CMECs were isolated and subjected to H/R. Pretreatment of F_2_ leads to a reduction in H/R injury, as evidenced by increased cell viability, decreased lactate dehydrogenase (LDH) leakage and apoptosis, together with enhanced AMP-activated protein kinase (AMPK) and liver kinase B1 (LKB1) phosphorylation in H/R ECs. Blockade of AMPK with compound C reversed F_2_-induced inhibition of H/R injury, as evidenced by decreased cell viability, increased LDH release and apoptosis. Moreover, compound C also blocked the ability of F_2_ to reduce H/R-induced reactive oxygen species (ROS) generation. Supplementation with the ROS scavenger N-acetyl-L-cysteine (NAC) reduced ROS levels, increased cell survival rate, and decreased both LDH release and apoptosis after H/R. In conclusion, our data indicate that F_2_ may mitigate H/R injury by stimulating LKB1/AMPK signaling pathway and subsequent suppression of ROS production in CMECs.

## INTRODUCTION

Myocardial ischemia/reperfusion (I/R) injury is a universal cardiovascular disease with major cause of morbidity, mortality all around the world and great cost to the society [1]. However, its pathogenesis remains obscure. Cardiac microvascular endothelial cells (CMECs) play a pivotal role in the development, contractile performance, and rhythmicity of heart [2]. It is well known that the endothelium, especially the microvascular endothelium, is highly susceptible to hypoxia and plays a crucial role during I/R injury [3]. During hypoxia/reoxygenation (H/R), endothelial cells exhibit a proinflammatory phenotype, including the induction of vasoconstrictive agents, leukocyte adhesion molecules and procoagulant factors [4]. Moreover, rats subjected to I/R injury manifest increased apoptosis of CMECs [5]. Therapeutic strategies focusing on the maintenance of endothelial cell function have been shown to alleviate myocardial injury [4, 6].

The heterotrimeric serine/threonine protein AMPK is composed of a catalytic kinase subunit (α) and two regulatory subunits (β and γ) [7]. There are a variety of isoforms of each subunit. The heterotrimers formed by different isoforms are not uniform in tissue and subcellular localization [8]. In mammalian cells, AMPK has been identified as a major energy sensor and plays a crucial role in cellular responses to energy-restricted conditions by switching on pathways that produce energy and shutting off those that consume it [9]. When the cellular AMP/ATP ratio elevates, AMPK is partly activated after binding to AMP and is totally activated when phosphorylated at the catalytic α-subunit by liver kinase B1 (LKB1), a tumor suppressor kinase [10]. LKB1/AMPK signaling has been shown to play a part in protection from apoptosis, specifically in response to conditions that increase the cellular AMP/ATP ratio [11]. It has been growingly recognized that activation of the AMPK pathway could confer cardioprotection against myocardial I/R injury [12–14].

A growing number of studies have shown that reactive oxygen species (ROS) -provoked oxidative stress plays a critical role in the development of myocardial I/R injury [15]. Excessive generation of ROS can cause cellular dysfunction and injury by directly oxidizing and damaging proteins, DNA and lipids, which ultimately result in cell death [16, 17]. Understanding how ROS producing and scavenging are modulated and developing strategies to decrease ROS production are significant for preventing I/R injury. An important signaling pathway implicated in ROS inhibition is the AMPK pathway. Recently, a plenty of studies have shown that activation of this pathway could decrease intracellular ROS levels [18, 19].

Haloperidol (Hal) is a classical antipsychotic drug and clinically used to treat the psychological diseases, including mania and schizophrenia. Hal has been shown to possess vascular relaxing activity and electrophysiological action. However, large sample investigations are limited by its extrapyramidal side effects. Thus, a series of Hal quaternary ammonium salt derivatives were designed and synthesized. One of these compounds, N-n-butyl haloperidol iodide (F_2_), was screened and found to preserve the cardiovascular activities without extrapyramidal adverse reactions. Our former investigations have shown that the cardioprotection of F_2_ may be linked to its capacity block calcium channels [20–22] and inhibition of the overexpression of early growth response gene-1, a major switch for multiple pathways of reperfusion injury [23–25]. Consistent with the attenuation of myocardial injury, we have also demonstrated that F_2_ inhibits H/R-induced ROS generation in cardiomyocytes [26]. However, related studies of F_2_ on cultured CMECs *in vitro* are limited. Whether the effects of F_2_ on H/R endothelial cells are associated with its antioxidant effect remains to be established. In this study, we aimed to investigate whether F_2_ reduces apoptosis and injury by activating the LKB1/AMPK pathway and the subsequent reduced ROS levels in primary cultured CMECs.

## RESULTS

### Characterization of cell type

**Figure 1:**
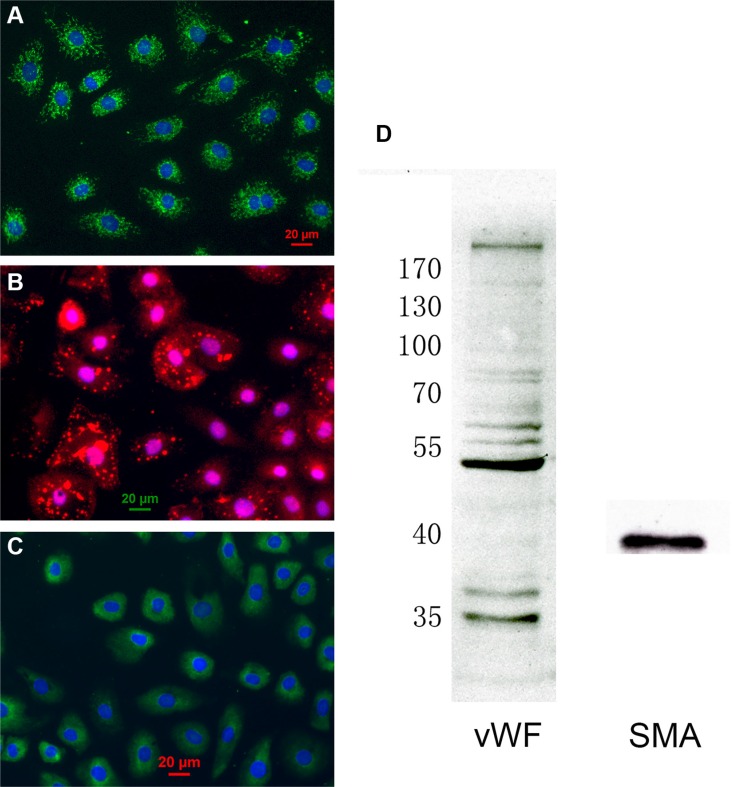
Characterization of cardiac microvascular endothelial cells (CMECs) (**A**) Cultured CMECs were immunostained for CD31 (green) and stained with DAPI for DNA (blue). (**B**) CMECs were immunostained for von Willebrand factor (red) and stained with DAPI for DNA (blue). (**C**) CMECs were immunostained for α-smooth muscle actin (green) and stained with DAPI for DNA (blue). (**D**) CMECs were lysed and probed for different proteins by western blot.

Primary cultures of CMECs stained positively for CD31 (Figure 1A) and von Willebrand factor (vWF), confirming their status as endothelial cells. In addition, CMECs stained positively for α-smooth muscle actin (SMA), a protein documented to be absent in endothelial cells of the larger vessels [27]. Immunocytochemistry for the endothelial cell marker vWF displayed a punctate distribution (Figure 1B), and SMA immunocytochemistry displayed a diffuse cytoplasmic staining with prominent cytoskeletal filaments (Figure 1C). Furthermore, both vWF and SMA were detected by Western blot (Figure 1D). These results confirm that our cells are CMECs.

### F_2_ attenuates H/R-induced CMEC death

**Figure 2:**
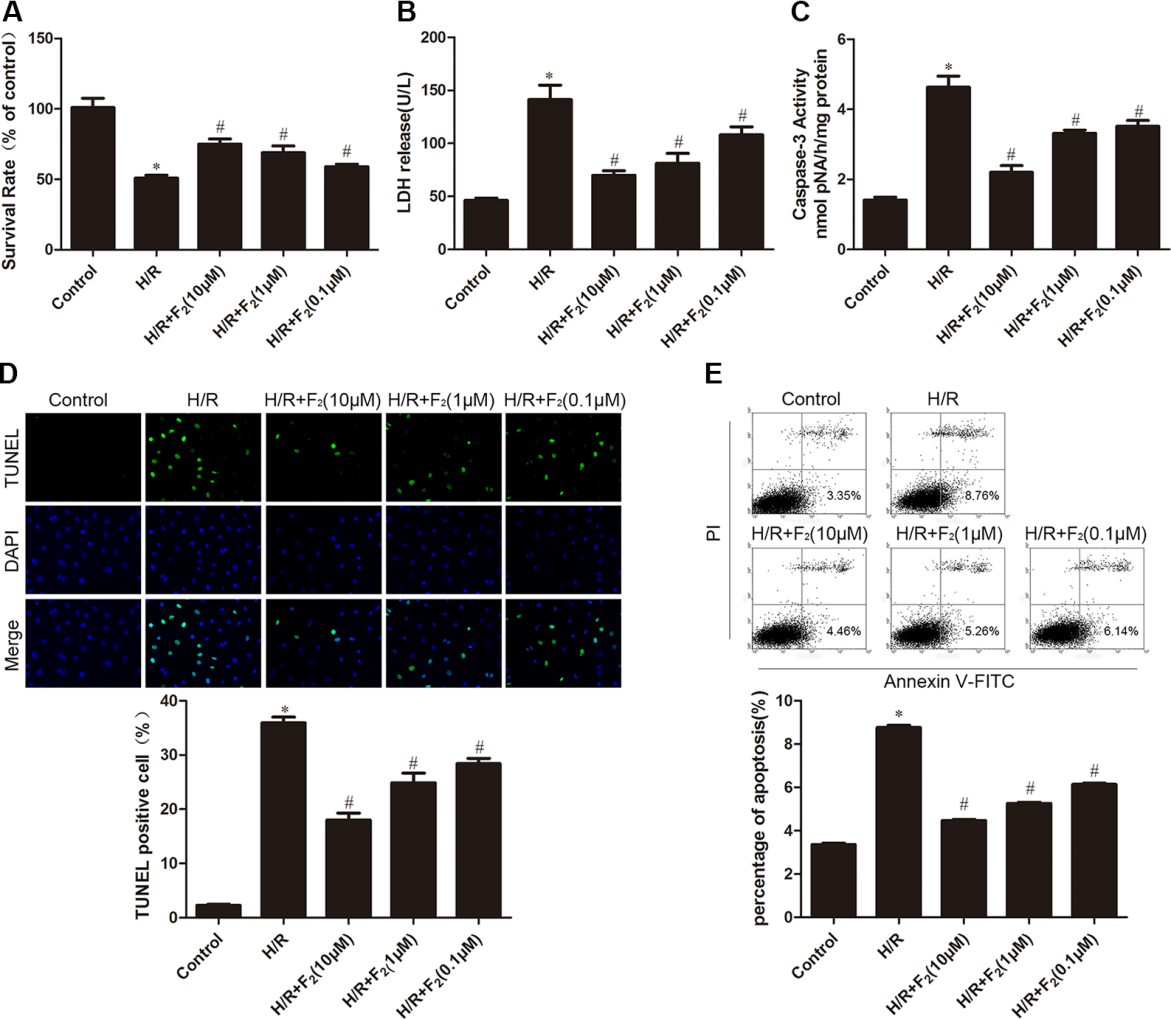
Effects of F_2_ on H/R-induced injury and apoptosis in CMECs (**A**) MTT assay was used to determine cell viability. (**B**) LDH leakage in culture medium at the end of reoxygenation was measured. (**C**) Caspase-3 activity in cell lysates was measured. (**D**) TUNEL assay for apoptosis. (**E**). Flow cytometry for apoptosis. The images are taken by 400 × magnification. All values are represented as means ± S.D confirmed in three separate experiments. ^*^P < 0.05 vs. control; ^#^*P* < 0.05 vs. H/R. H/R: hypoxia/reoxygenation.

Exposure of CMECs to H/R resulted in a significant decline in cell viability, while F_2_ treatment dose-dependently increased the survival rate of endothelial cells experiencing H/R challenge (Figure 2A), with maximal protection occurring at 10 μM F_2_. Since the leakage of lactate dehydrogenase (LDH) is well known to be a marker of cellular injury, endothelial cell damage was evaluated by measuring LDH activity in culture medium. LDH leakage increased after H/R, but was markedly decreased by F_2_ treatment (Figure 2B).

### F_2_ suppresses H/R-induced CMEC apoptosis

We next determined the effects of F_2_ on H/R-provoked apoptosis by flow cytometric analysis and terminal deoxyuncleotidyl transferase-mediated dUTP nick end-labeling (TUNEL) assay. As shown in Figure 2C and 2D, H/R led to a significant increase in the apoptotic index; however, treatment of F_2_ markedly inhibited the apoptosis in CMECs subjected to H/R. Additionally, while caspase-3 activity, a critical stimulator of cell apoptosis, was significantly elevated after H/R, this H/R-evoked caspase-3 activation was suppressed by F_2_ (Figure 2E).

### F_2_ activates LKB1/AMPK in CMECs

**Figure 3:**
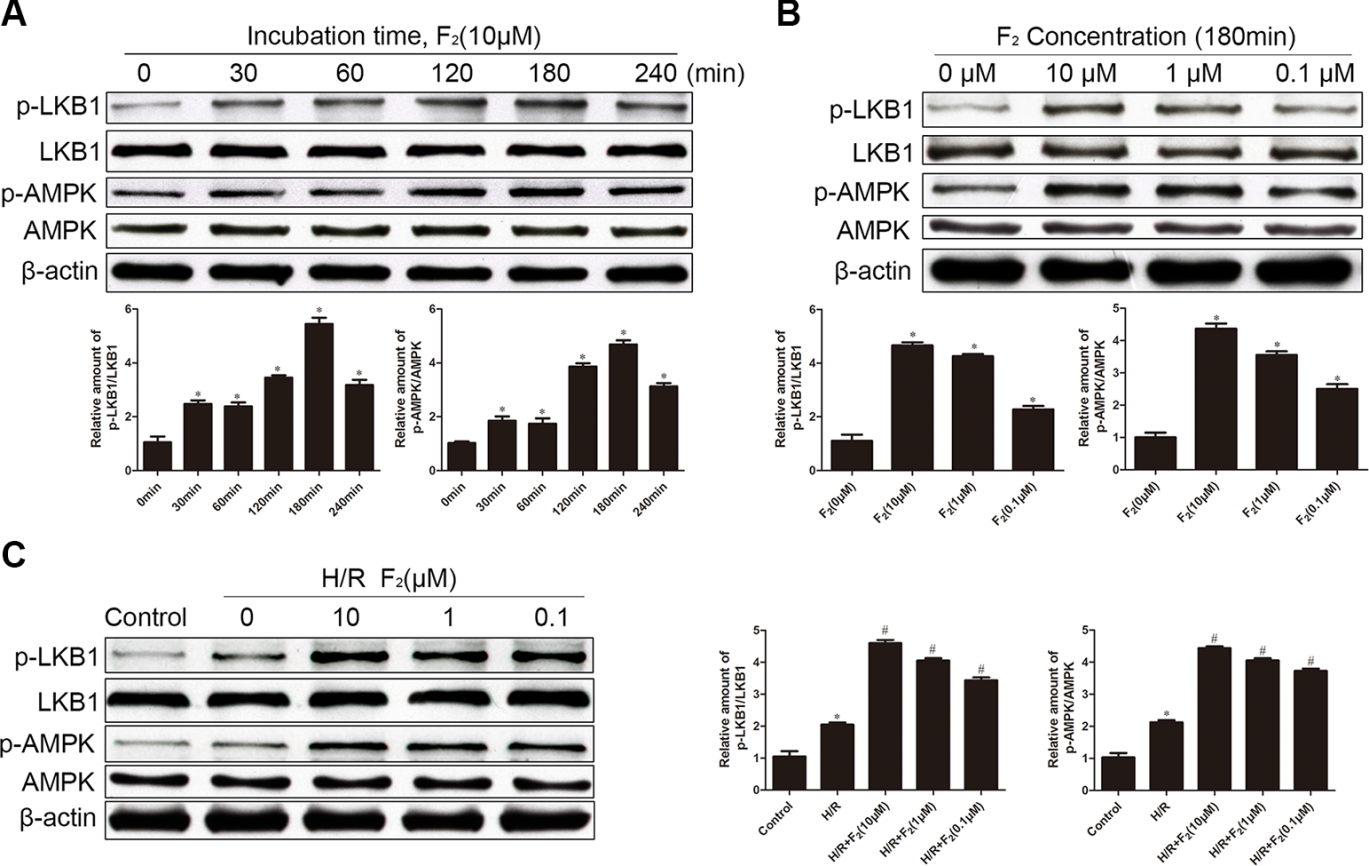
Effects of F_2_ on phosphorylation of LKB1 and AMPK in CMECs, as assessed by western blot (**A**) Time-dependent changes in P-LKB1 and P-AMPK after stimulation with F_2_. (**B**) Dose-dependent changes in P-LKB1 and P-AMPK after stimulation with F_2_. (**C**). P-LKB1 and P-AMPK in CMECs treated with F_2_ after H/R. All values are represented as mean ± S.D confirmed in three separate experiments. ^*^P < 0.05 vs. control; ^#^*P* < 0.05 vs. H/R. H/R: hypoxia/reoxygenation.

Because AMPK reportedly protects endothelial cells from apoptosis and hypoxic injury [28], we assessed the level of activated (phospho-) AMPK after H/R treatment. H/R increased the phosphorylation of AMPK in the control group, but F_2_ dose-dependently enhanced this induction (Figure 3A). In parallel, F_2_ dose-dependently increased the phosphorylation of LKB1, an upstream kinase of AMPK in endothelial cells. We next assessed the phosphorylation of LKB1 and AMPK in CMECs after treatment with F_2_ or vehicle. F_2_ time-dependently stimulated the phosphorylation of LKB1 and AMPK, with maximal levels occurring at 180 min (Figure 3B). Moreover, F_2_ could stimulate the phosphorylation of LKB1 and AMPK in a dose-dependent manner (Figure 3C).

### AMPK participates in the protective effects of F_2_ on H/R injury in CMECs

**Figure 4:**
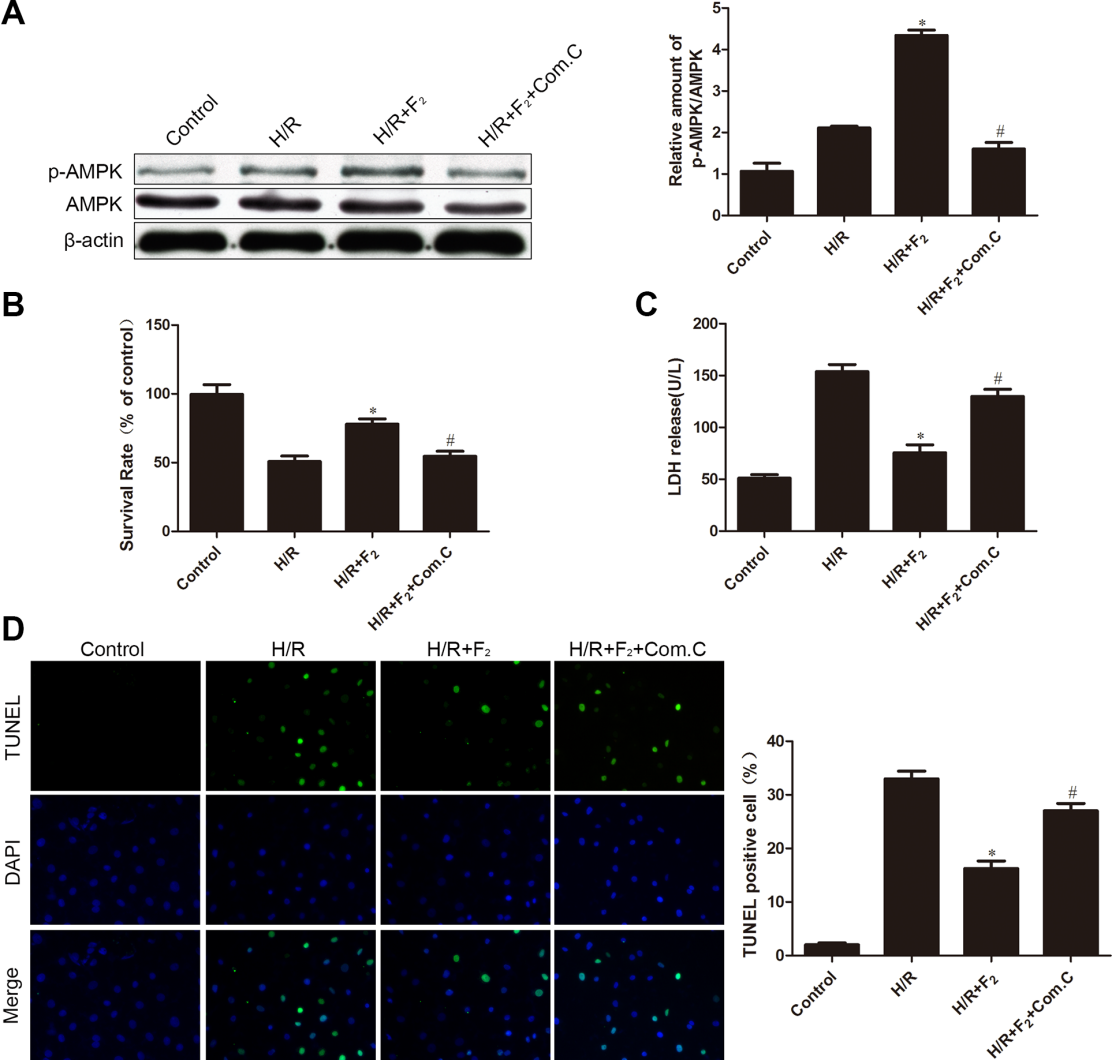
Influence of AMPK inhibitor compound C on F_2_-mediated phosphorylation of AMPK and H/R injury (**A**) P-AMPK/AMPK levels were analyzed by western blot. (**B**) Cell viability was determined by MTT assay. (**C**) LDH activity in culture medium was measured. (**D**) TUNEL assay for apoptosis. The images are taken by 400 × magnification. All values are represented as mean ± S.D confirmed in three separate experiments. ^*^P < 0.05 vs. H/R; ^#^*P* < 0.05 vs. H/R + F_2_. H/R: hypoxia/reoxygenation.

To examine whether AMPK is involved in F_2_-mediated protection against H/R damage, we used the AMPK inhibitor compound C. Pretreatment of compound C significantly reduced the F_2_-mediated increase in AMPK phosphorylation in the H/R-challenged endothelial cells (Figure 4A). Compound C also abrogated the F_2_-induced increase in cell survival rate and F_2_-induced decrease in both LDH release and TUNEL-positive cells in the H/R- induced endothelial cells subjected to H/R (Figure 4B–4D). Thus, F_2_ can reduce H/R injury partly through an AMPK signaling pathway.

### F_2_ inhibits ROS generation in CMECs after H/R

**Figure 5:**
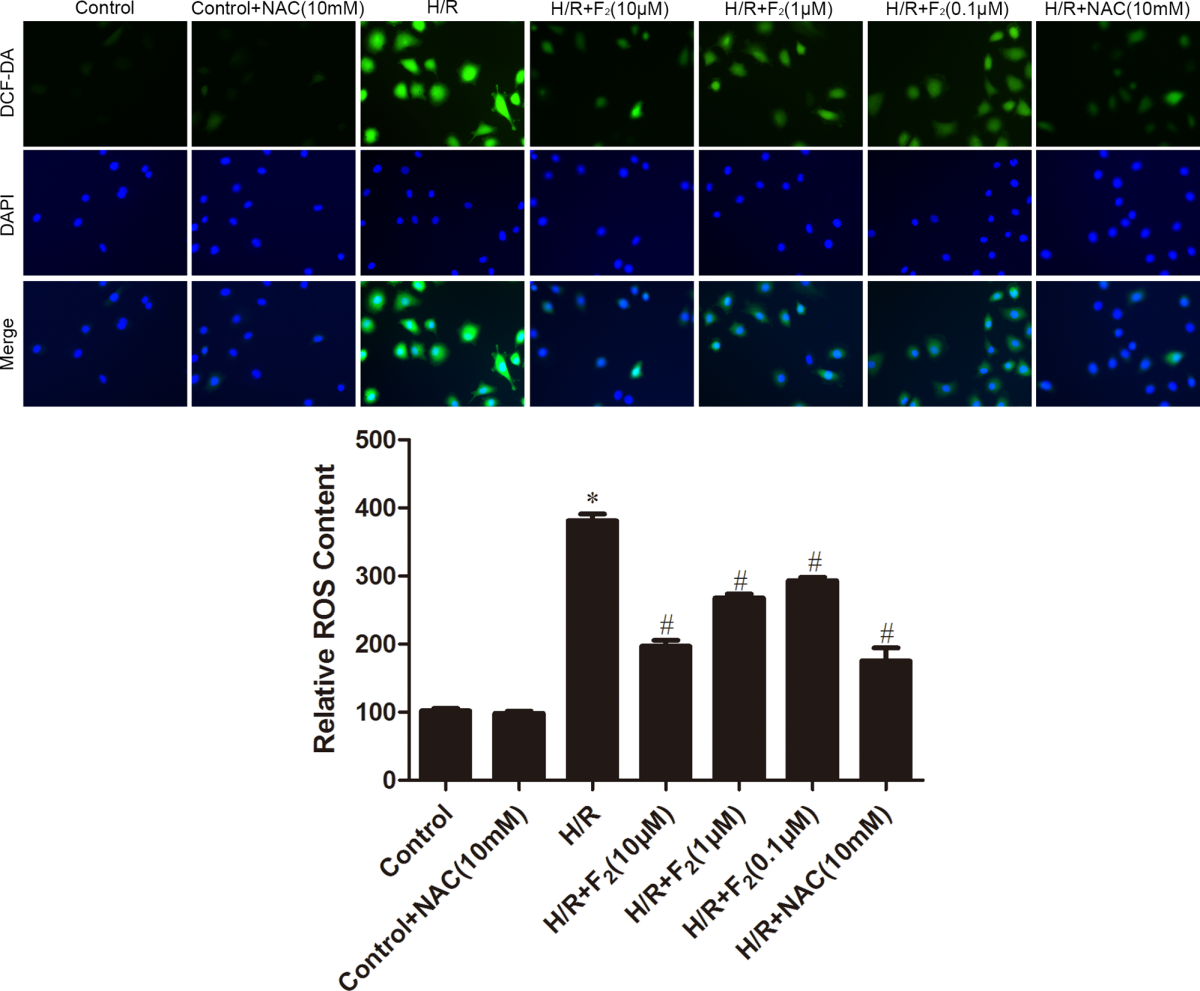
Effects of F_2_ and NAC on ROS levels in CMECs after H/R, as assessed by DCFH-DA staining The images are taken by 400 × magnification. All values are represented as mean ± S.D confirmed in three separate experiments. ^*^P < 0.05 vs. control; ^#^*P* < 0.05 vs. H/R. H/R: hypoxia/reoxygenation.

By DCFH-DA staining, H/R increased intracellular ROS generation in CMECs, while treatment of F_2_ reduced ROS production in a dose-dependent manner (Figure 5). Although N-acetyl-L-cysteine (NAC), a classical ROS scavenger, had no effects on ROS levels under normoxic conditions, it completely prevented H/R-induced ROS generation (Figure 5).

### Treatment with the ROS scavenger NAC attenuates H/R injury in CMECs

**Figure 6:**
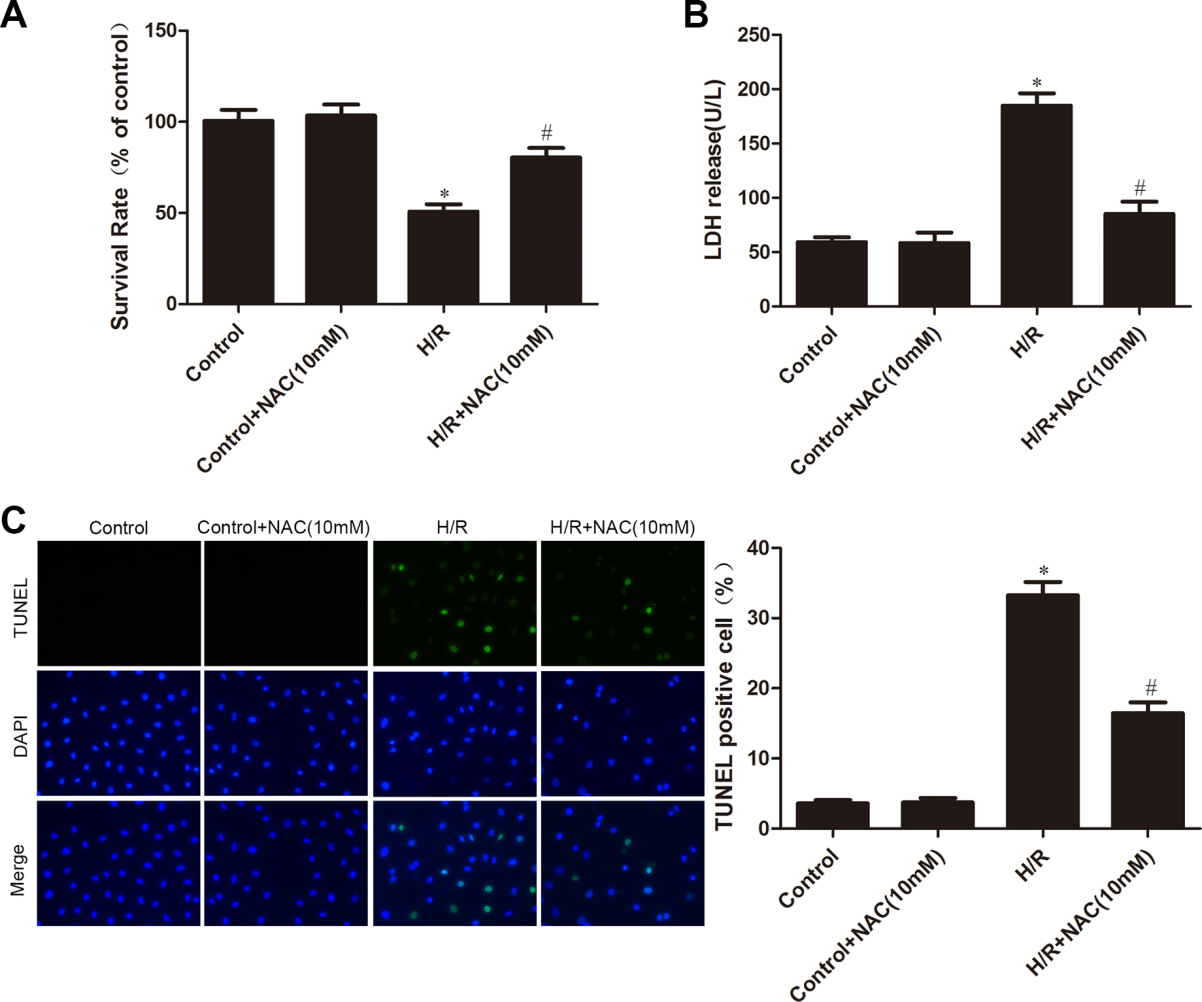
Effects of NAC on H/R injury (**A**) MTT assay was used to measure cell viability. (**B**) LDH leakage in culture medium was determined. (**C**) TUNEL assay for apoptosis. The images are taken by 400 × magnification. All values are represented as mean ± S.D confirmed in three separate experiments. ^*^P < 0.05 vs. control; ^#^*P* < 0.05 vs. H/R. H/R: hypoxia/reoxygenation.

To determine whether ROS mediated H/R-induced changes in cell survival, LDH leakage and apoptosis, we pre-treated cells with the ROS scavenger NAC. Figure 6A shows that NAC had no influence on control cells, but inhibited the H/R-induced decrease in cell survival. Furthermore, NAC also decreased H/R-induced LDH leakage and apoptosis (Figure 6B and 6C).

### F_2_ reduces ROS levels via activating AMPK pathway

**Figure 7:**
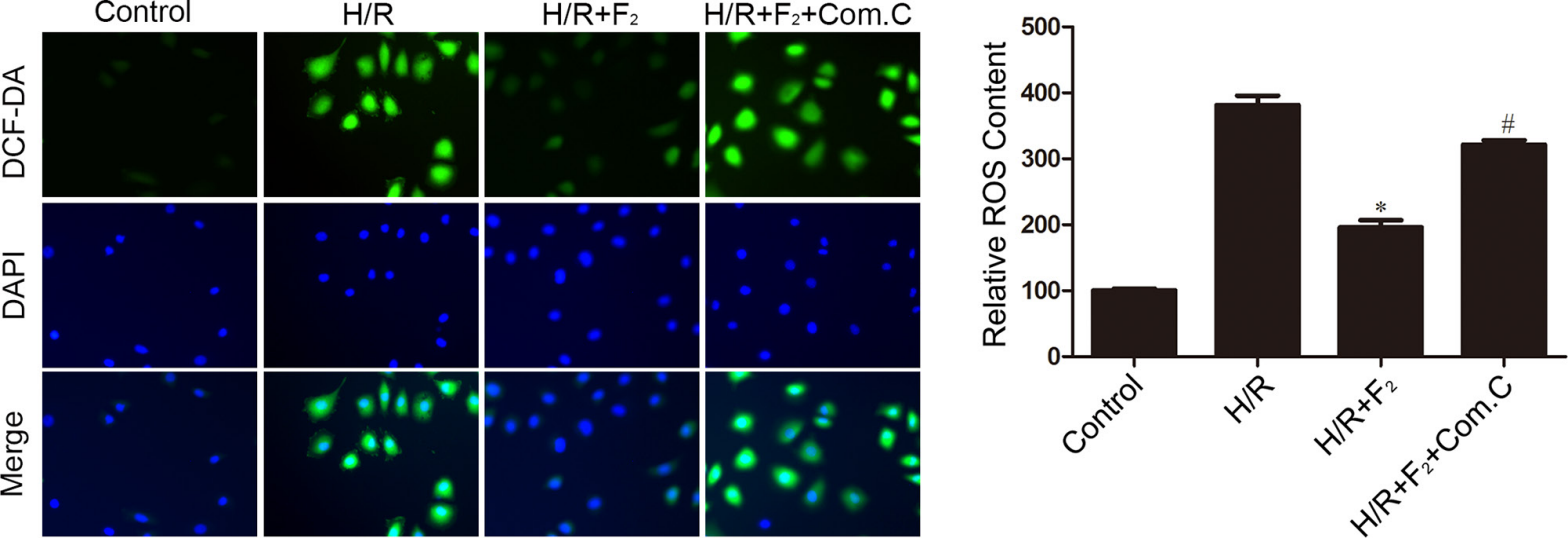
Influence of AMPK inhibitor compound C on F_2_-mediated decreases in ROS levels in CMECs after H/R, as assessed by DCFH-DA staining The images are taken by 400 × magnification. All values are represented as mean ± S.D confirmed in three separate experiments. ^*^P < 0.05 vs. H/R; ^#^*P* < 0.05 vs. H/R + F_2_. H/R: hypoxia/reoxygenation.

To ascertain whether the effect of F_2_ on oxidative stress was relative to the activation of AMPK, the effect of compound C on ROS production was investigated. As illustrated in Figure 7, H/R treatment increased ROS levels. Treatment with F_2_ markedly reduced ROS production following H/R treatment. This anti-oxidative effect was virtually abolished by cotreatment with compound C. These results demonstrate that F_2_ attenuates H/R-induced oxidative stress by activating AMPK.

## DISCUSSION

CMECs are sensitive to hypoxic injury, which influences the prognosis, development, and pathogenesis of cardiovascular disease [29]. It has been formerly documented that endothelial dysfunction precedes cardiomyocyte damage in I/R conditions [30]. Attenuating endothelial injury can eventually reduce cardiomyocyte death and apoptosis after I/R. Presently, we found that treatment of CMECs with F_2_ increased cell viability and decreased LDH release in a dose-dependent manner, providing the direct evidence that F_2_ could mitigate cardiac microvascular endothelial H/R injury.

The pathogenesis of I/R-evoked myocardial injury is evidently multifactorial. Limitation of apoptosis is identified as an important therapeutic approach for ischemic heart disease [31, 32]. We demonstrated in this study that F_2_ dose-dependently suppressed apoptosis in CMECs subjected to H/R injury as evidenced by TUNEL assay, flow cytometric analysis and downregulated caspase-3 activity. Similar studies in cultured neonatal rat cardiomyocytes also showed that F_2_ exerted an anti-apoptotic effect against H/R injury via inhibiting protein kinase α [33]. This suggests that the key role for F_2_ in endothelial H/R injury probably is its anti-apoptotic role.

The mechanism by which F_2_ protects CMECs against H/R injury has been incompletely understood; some evidence reveals that preventing an increase in oxidative stress is involved [23]. Our results showed that F_2_ treatment activated the AMPK pathway in untreated CMECs or CMECs under H/R, which is an important finding. Studies have documented that AMPK exerts a cardioprotective effect during I/R by increasing glucose transporter translocation, inhibiting apoptosis, and ultimately reducing the myocardial infarction size [34]. In the current study, compared with the F_2_-treated group, combined treatment of F_2_ and compound C significantly decreased cell viability, increased LDH leakage and apoptosis, which suggests that F_2_ attenuates H/R injury and apoptosis at least in partially through AMPK activation.

During myocardial I/R injury, the generation of adenosine triphosphate is reduced due to limited oxygen supply, which enhances mitochondrial oxidative phosphorylation dysbolism and glycolysis, the major changes to produce a lot of H^+^, NADH^+^, Ca^2+^, and lactic acid [35]. These contribute to mitochondrial dysfunction and the succeeding ROS accumulation [36]. The Increased ROS levels in the vasculature results in endothelial damage [37], which acts as the major etiological factor underlying I/R injury [38]. We showed in our former study that F_2_ reduces the production of ROS after H/R in cardiomyocytes, which is accompanied by improved myocardial function [26]. Consistent with these findings, our present data demonstrated that F_2_ dose-dependently suppresses the generation of ROS in H/R-challenged CMECs. Importantly, pretreatment with the ROS scavenger NAC markedly attenuates H/R injury, as evidenced by increased cell viability, reduced LDH release and apoptosis. Together, these data suggest that F_2_ protects CMECs from H/R injury through its ability to suppress oxidative stress.

The signals by which F_2_ inhibits H/R-induced excessive ROS are not fully characterized. A promising signaling pathway associated with ROS modulation is the AMPK pathway. Recently, Zheng et al. [39] reported that in retinal endothelial cells, under hyperglyceamic situations, metformin activates the LKB1/AMPK pathway to inhibit ROS generation. In this study, compared with the F_2_-treated group, combined treatment of F_2_ and compound C significantly increased ROS levels, which suggests that suppression of ROS generation by F_2_ at least partly via AMPK activation. In summary, improved efficacy with the usage of F_2_ in protecting against H/R-induced apoptosis and injury can possibly be attributed to the activation of LKB1/AMPK pathway and the subsequent inhibition of ROS generation in CMECs, which might be an important mechanism by which it alleviates myocardial I/R injury. The findings suggest the potential therapeutic value of F_2_ in the prevention and rescue for various cardiovascular diseases caused by oxidative stress.

## MATERIALS AND METHODS

### Materials and antibodies

The following rabbit polyclonal primary antibodies were purchased from Santa Cruz Biotechnology (CA, USA): phospho-LKB1 antibody, LKB1 antibody, phosphor-AMPK antibody, and AMPK antibody. Mouse monoclonal β-actin was bought from Boster Biological Engineering (Wuhan, China). Anti-mouse and anti-rabbit secondary antibodies were bought from Beyotime Biotecnology (Nantong, China). Endothelial cell growth supplements (ECGS) and compound C were purchased from Millipore (CA, USA). The ROS scavenger N-acetyl-L-cysteine (NAC) and 2′, 7′-dichlorofluorescein acetyl acetate (DCFH-DA) were purchased from Sigma Chemical Co. (St. Louis, USA). Other reagents and chemical were from local commercial corporations. F_2_ and compound C were dissolved in dimethyl sulfoxide (DMSO). The final DMSO concentration was ≤ 0.1%. The concentration had no influence on viability of normal primary CMECs and H/R-induced primary CMECs.

### Animals

Adult Sprague-Dawley rats (250–300 g) were purchased from the Laboratory Animal Center (Shantou, China). Care of rats in this study cohered with the Guide for the Care of Use of Laboratory Animals (NIH Publication, 1996). All experiments were carried out in conformity with the guidelines for the Principles of Laboratory Animal Care and Use of Laboratory Animals (NIH publication, 2011). This study was approved by the Institutional Animal Care and Use Committee of Shantou University Medical College.

### Isolation and culture of CMECs

CMECs were isolated from the left ventricles of hearts from neonatal rats, as previously described [40], with slight modifications. Briefly, the left ventricles were finely minced and digested with 0.1% trypsin for twice, for 5 min at 37°C each time, in a shaking water bath. Dissociated cells were then filtered and centrifuged at 1000 *g* for 5 min. Cells were resuspended in DMEM supplemented with 10% FBS, 15 μg/ml ECGS, 100 μg/ml streptomycin, 100 U/ml penicillin, 40 U/ml heparin and plated on 2% gelatin-coated dishes.

### Hypoxia/reoxygenation procedure and experimental protocols

To induce hypoxic stress, the normal culture medium was substituted for pH 6.2 buffer composing of 137 mM NaCl, 4 mM HEPES, 0.49 mM MgCl_2_, 12 mM KCl, 0.9 mM CaCl_2_·H_2_O, 20 mM Na lactate. Endothelial cells were placed in an air-tight chamber saturated with pure N_2_ for 4 h at 37°C. The buffer was then exchanged with normal culture medium, and the cells were cultured in a normoxic incubator for 6 h of reoxygenation. CMECs at passage 3–5 were used in this study. After 24 h synchronization, endothelial cells were randomly divided into seven groups for treatment: control (incubated under normal condition); H/R; H/R pretreated with F_2_ (10 μM), F_2_ (1 μM), F_2_ (0.1 μM), or NAC (10 mM); H/R pretreated with compound C (5 μM) and F_2_ (10 μM).

### Cell viability assay

Cell viability of CMECs was determined by MTT assay as previously depicted. Cells (1 × 10^5^/ml) were plated into 96-well plates. After different treatments, each well was administered with 20 μl MTT (5 g/L, Sigma) and then incubated at 37°C for 4 h. The medium was carefully discarded and the formed formazan was dissolved in 200 μl DMSO. After shaking for 15min at room temperature in the dark, absorbance at 490 nm was photometrically determined.

### Measurement of LDH activity

The leakage of LDH was determined spectrophotometrically by using an assay kit from Jiancheng Bioengineering Institute (Nanjing, China). Briefly, after H/R treatment, 50 μl of culture supernatant was carefully aspirated and saved for LDH measurement. After successive addition of several kinds of reaction buffer, the absorbance at 440 nm was measured by using a spectrophotometer.

### TUNEL assay for apoptosis

CMECs were grown on gelatin-coated coverslips and fixed with 4% glutaraldehyde. The TUNEL assay was involved in use of the Cell Death Detection Kit (Promega, USA). Staining was observed by using a fluorescence microscope (Olympus, Japan). Apoptosis was expressed as the ratio of positive nuclei/total nuclei from five randomly chosen fields.

### Flow cytometric detection of apoptosis

Early apoptosis was identified by the Annexin V-FITC apoptosis detection kit (Biolegend, USA) in conformity to the manufacturer's instructions. In short, cells were harvested, rinsed twice with PBS, and then incubated with 5 μl Annexin V-FITC and 10 μl propidium iodide working solution (100 μg/ml) for 15 min in the dark at room temperature. The apoptosis of each sample was then detected by use of a FACSort flow Cytometer (Becton Dickinson, USA). About 10,000 cells were recorded in each sample, and the results were analyzed by use of WinMDI2.9 software.

### Measurement of caspase-3 activity

Caspase-3 activity was determined by a caspase-3 assay kit from Keygen Biotechology (Nanjing, China) in accordance with the manufacturer's directions. CMECs were harvested by centrifugation and rinsed twice with PBS. Cell number was then adjusted to 2 × 10^6^. Each sample was administrated with 100 μl lysis buffer and incubated on ice for 30 min. Lysates were centrifuged at 12000 g for 10 min and clear supernatants were administrated with 2 mM caspase-3 substrate (Ac-DEVD-pNA) for 4 h in a 37°C incubator, and then the absorbance was detected with a spectrophotometer at the wavelength of 405 nm. The enzymatic activity of caspase-3 in cell lysates was directly relative to the color reaction. The BCA method was utilized to quantify protein concentration.

### Western blot analysis

After different treatment as indicated, CMECs were harvested, scraped off in lysis buffer and centrifuged for 15 min at 12000 g at 4°C. The protein concentration was measured using a BCA assay kit (Pierce, USA). Sample aliquots (usually 100 μg) were boiled for 5 min and equal protein amounts (usually 30 μg) were separated by 10% SDS-PAGE. Proteins were then transferred to a nitrocellulose membrane. Blots were blocked and immunoblotted with anti-LKB1, phospho-LKB1, AMPK, phospho-AMPK and β-actin antibodies (typically 1:2000 dilution) at 4°C overnight, followed by HRP-conjugated secondary antibodies (typically 1:2000 dilution) for 1 h at room temperature. Transferred proteins were detected using a SuperSignal detection kit (Pierce, USA), and the results were analyzed with Gel-Pro Image Analysis Software.

### Detection of intracellular ROS generation

Intracellular oxidants in CMECs were determined by probing with dichlorodihydrofluorescin diacetate (DCFH-DA). Briefly, cells were incubated with 10 μM of DCFH-DA for 30 min, and then treated with DAPI (1 μM) for 10 min. Cells were rinsed twice with PBS, and images were captured from six or more randomly chosen fields using a fluorescence microscope (Olympus, Japan).

### Statistical analysis

All values are represented as mean ± S.D. Statistical analysis of the data was performed using one-way analysis of variance followed by the Newman-Keuls test. A value of *P* < 0.05 was considered as statistically significant.

## Abbreviations

AMPK: AMP-activated protein kinase
CMECs: cardiac microvascular endothelial cells
DMSO: dimethyl sulfoxide F2, N-n-butyl haloperidol iodide
H/R: hypoxia/reoxygenation
I/R: ischemia/reperfusion
LDH: lactate dehydrogenase
LKB1: liver kinase B1
MTT: 3-(4,5-dimethylthiazol-2-yl)-2,5-diphenyltetrazolium bromide
NAC: N-acetyl-L-cysteine
ROS: reactive oxygen species
TUNEL: terminal deoxyuncleotidyl transferase-mediated dUTP nick end-labeling
